# Pulmonary complications associated with veno-arterial extra-corporeal membrane oxygenation: a comprehensive review

**DOI:** 10.1186/s13054-020-02937-z

**Published:** 2020-05-11

**Authors:** Aurélien Roumy, Lucas Liaudet, Marco Rusca, Carlo Marcucci, Matthias Kirsch

**Affiliations:** 1 Department of Cardiovascular Surgery, University Hospital, Lausanne, Switzerland; 2 Department of Intensive Care Medicine, University Hospital, Lausanne, Switzerland; 3 Department of Anesthesiology, University Hospital, Lausanne, Switzerland

**Keywords:** Extracorporeal membrane oxygenation, Cardiogenic shock, Lung injury

## Abstract

Veno-arterial extracorporeal membrane oxygenation (VA-ECMO) is a life-saving technology that provides transient respiratory and circulatory support for patients with profound cardiogenic shock or refractory cardiac arrest. Among its potential complications, VA-ECMO may adversely affect lung function through various pathophysiological mechanisms. The interaction of blood components with the biomaterials of the extracorporeal membrane elicits a systemic inflammatory response which may increase pulmonary vascular permeability and promote the sequestration of polymorphonuclear neutrophils within the lung parenchyma. Also, VA-ECMO increases the afterload of the left ventricle (LV) through reverse flow within the thoracic aorta, resulting in increased LV filling pressure and pulmonary congestion. Furthermore, VA-ECMO may result in long-standing pulmonary hypoxia, due to partial shunting of the pulmonary circulation and to reduced pulsatile blood flow within the bronchial circulation. Ultimately, these different abnormalities may result in a state of persisting lung inflammation and fibrotic changes with concomitant functional impairment, which may compromise weaning from VA-ECMO and could possibly result in long-term lung dysfunction. This review presents the mechanisms of lung damage and dysfunction under VA-ECMO and discusses potential strategies to prevent and treat such alterations.

## Introduction

Veno-arterial extracorporeal membrane oxygenation (VA-ECMO) is a life-saving technology providing respiratory and circulatory support in patients with refractory cardiogenic shock or cardiac arrest [1] and which may give time to plan future therapeutic decisions such as the insertion of long-term cardiac assist devices or heart transplantation (HTX) [2]. Notwithstanding its potential benefits, VA-ECMO remains associated with significant morbidity and mortality [1]. This is partly due to the patients’ critical condition, but also to complications related to VA-ECMO, notably renal failure, sepsis, bleeding, thromboembolism, limb ischemia, and multi-organ failure [3, 4].

VA-ECMO-induced pulmonary complications are much less recognized, except from the pulmonary congestion related to left ventricle pressure overload induced by retrograde VA-ECMO flow within the thoracic aorta [5]. Beside this particular aspect, several additional mechanisms may contribute to lung damage and dysfunction in the setting of VA-ECMO. The latter may be assimilated to a simplified cardiopulmonary bypass (CPB) circuit, and both techniques share common pitfalls with respect to lung physiology. CPB may alter pulmonary function after cardiac surgery by promoting an inflammatory response via biomaterial-dependent and biomaterial-independent factors, the collapse of lungs during the procedure, the shunting of pulmonary circulation, and the phenomenon of lung reperfusion injury which takes place once CPB is weaned [6]. During VA-ECMO, although such processes are attenuated, they still occur at different stages of support and at various degrees, and they may persist for days or weeks. In such conditions, the combination of a chronic inflammatory response, pulmonary congestion, and lung ischemia could foster a wealth of morphological and functional alterations which could interfere with patient’s recovery and compromise the overall planned therapeutic strategy. In this review, we discuss the pathophysiological mechanisms and potential clinical implications of the pulmonary complications associated with VA-ECMO.

## VA-ECMO-related systemic inflammatory response syndrome

The induction of a systemic inflammatory response syndrome (SIRS) by the contact of blood with biomaterial is a typical consequence of extracorporeal circulation [7]. While extensively studied in the field of CPB, this is also witnessed during VA-ECMO, as recently reviewed [8]. In addition, patients undergoing VA-ECMO are critically ill and suffer from profound cardiogenic shock, which by themselves contribute to the development of SIRS [9]. The lung is a major target of inflammatory injury in the context of SIRS, owing to its extensive capillary bed and the presence of abundant immune cells within the lung parenchyma. Therefore, the occurrence of SIRS in the context of ECMO provides a highly favorable environment for the development of acute lung injury [10, 11]. The main mechanisms triggering the inflammatory response to biomaterials are presented below.

### Humoral cascades

**Fig. 1:**
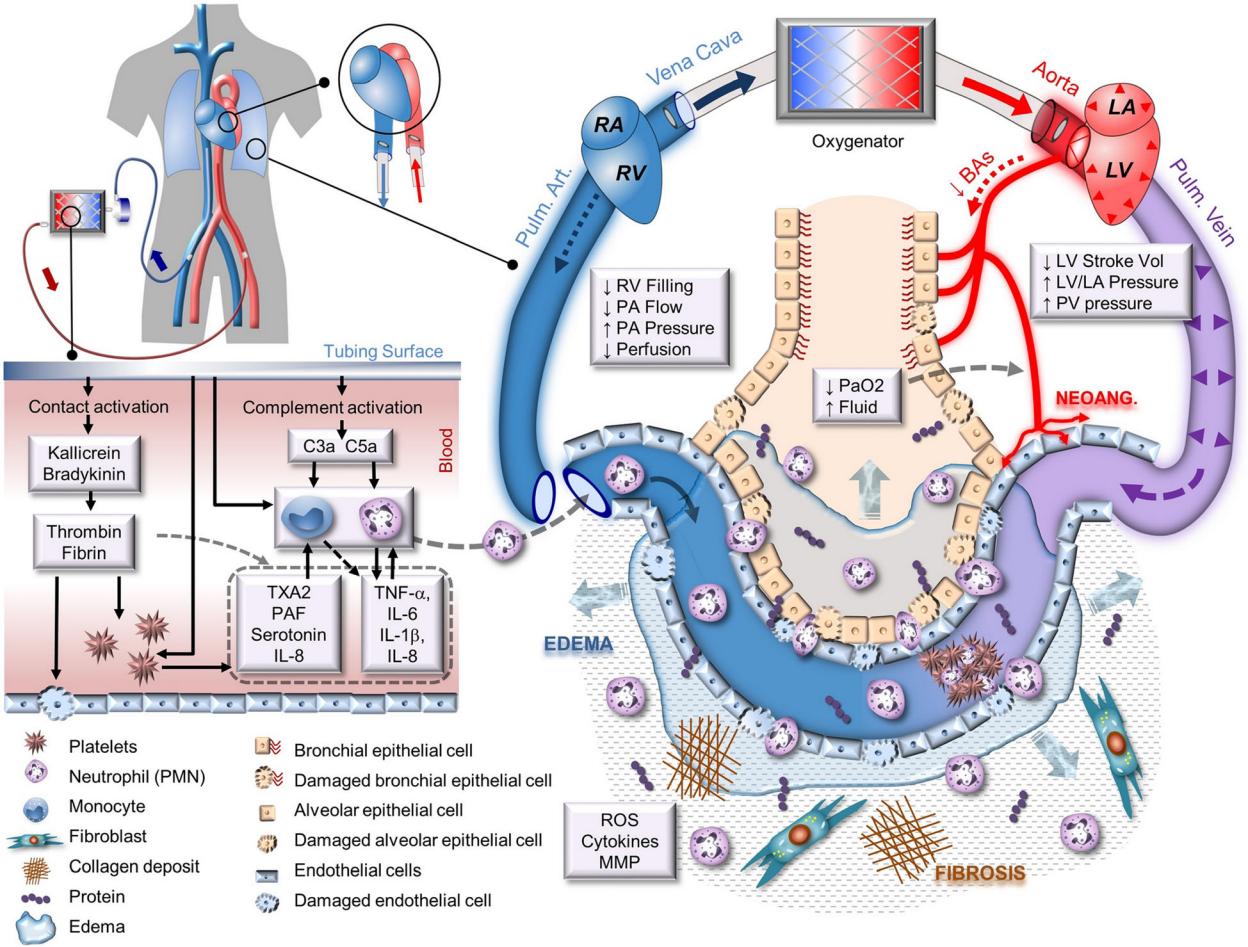
Main VA-ECMO-induced mechanisms of lung damage and dysfunction. Left side: SIRS is initiated by the blood contact with the circuitry surface. It activates humoral cascades, platelets, and leukocytes, leading eventually to EC injury and activated PMN sequestration into the lung parenchyma. Right side: EC injury favors fluid infiltration into both alveolar space and lung parenchyma, leading to pulmonary edema, which is aggravated by the increase of pulmonary vein pressure. Alveolar edema and decreased pulmonary artery perfusion lead to lung parenchymal ischemia which in turn maintains chronic inflammation and promotes neoangiogenesis and fibrosis generation

Blood contact with VA-ECMO circuitry activates the contact system (CS) and the complement system (Fig. 1). CS generates kallikrein [12], which activates monocytes and polymorphonuclear cells (PMNs), and triggers the intrinsic coagulation cascade, resulting in the rapid generation of thrombin and fibrin within the systemic circulation [13]. Thrombin activates platelets and endothelial cells (ECs) and induces the secretion of pro-inflammatory mediators and growth factors, such as interleukin-6 (IL-6), interleukin-8 (IL-8), or platelet-derived growth factor (PDGF) [13]. The extrinsic coagulation pathway is activated to a lesser extent, mainly through the release of tissue factor (TF) by activated monocytes and ECs. CS also generates bradykinine [12], which activates ECs and leukocytes, and elicits hemodynamic alterations including systemic vasodilation and pulmonary vasoconstriction [14]. Complement activation occurs via the alternative pathway, generating the anaphylatoxins C3a and C5a, which activate ECs. C5a is also a potent mediator of leukocyte chemotaxis. A peak of complement activation occurs within 1–2 h of ECMO onset, followed by a progressive decreases over the next 2 to 3 days [15].

The humoral response triggered by blood-biomaterial interaction comprises the release of multiple cytokines [16]. Whereas a balance between pro- and anti-inflammatory cytokines is reached several hours after VA-ECMO initiation [17], an initial imbalance in favor of pro-inflammatory TNF-α, IL-1β, and IL-6 leads to the activation of ECs and promotes the release of multiple inflammatory proteins by the liver such as fibrinogen, complement, and C-reactive protein. TNF-α plays a major role in the amplification of the early inflammatory response, by upregulating pro-inflammatory cytokines and prostaglandin synthesis, activating PMNs and ECs, and stimulating reactive oxygen species (ROS) production [18, 19].

### Cell activation

Platelets are activated by contact with the tubing surface and by thrombin and complement. Activated platelets foster the generation of pro-inflammatory cytokines, thromboxane A2 (TXA2), platelet-activating factor (PAF), P-selectin, and serotonin. TXA2 induces ECs activation and local vasoconstriction, while serotonin and P-selectin promote PMN-endothelial interactions [20]. Platelet activation is maximal at the initiation of VA-ECMO and progressively decreases over hours to days but remains persistent [21]. EC activation leads to their detachment from the basal membrane and disassembly of tight junctions, increasing vascular permeability with the development of sub-endothelial edema [22]. Moreover, activated ECs display an upregulated expression of adhesion molecules favoring PMN adhesion and transendothelial migration [23], and they also release cytokines, tissue factor, and ROS. Circulating PMNs, monocytes, and macrophages are spontaneously activated by tubing surfaces [24]. Furthermore, PMNs are activated by complement, histamine, serotonin, and PAF, which facilitate their adhesion to ECs, diapedesis, tissue infiltration [25], and the release of cytotoxic mediators, including proteases, cytokines, and ROS.

### Modulation of SIRS during VA-ECMO

At variance with CPB, VA-ECMO is generally maintained over several days. The initial significant SIRS gradually decreases [15, 17], mostly through the progressive build-up of counter-regulatory mechanisms leading to compensatory anti-inflammatory response and of possible biomaterial inactivation [26]. Still, a delayed persisting inflammatory response can be observed several days after VA-ECMO implementation, whose underlying mechanisms may involve the presence of low concentration of endotoxin within the circulation, which may sustain complement activation, cytokine release, and ROS generation, to elicit a sepsis-like inflammation [27, 28]. The low-level inflammatory response induced by pulmonary low flow is another potential mechanism (see below).

### Strategies to reduce SIRS-induced lung damages during VA-ECMO

Some potential therapies have been proposed to downregulate inflammation and possibly improve lung outcome in this setting. The replacement of a silicon oxygenator by a poly-methyl pentene oxygenator has been associated with reduced radiological signs of pulmonary inflammation on chest X-ray [29], while the administration of steroids in patients undergoing VA-ECMO has been associated with shortened mechanical ventilation time, although without any survival benefit [30].

## VA-ECMO-related pulmonary congestion

### Pathophysiology

Peripheral (femoro-femoral) VA-ECMO provides a non-physiological blood flow promoting significant hemodynamic perturbations (Fig. 1). The retrograde reinjection of blood into the thoracic aorta increases LV afterload and impedes aortic valve opening, while increasing myocardial oxygen demand [31]. In the setting of cardiogenic shock, these disturbances may worsen LV performance and dramatically reduce LV stroke volume [31, 32]. In addition, if LV residual function is insufficient to permit aortic valve opening, progressive LV distension will occur, due to persisting venous return through pulmonary and bronchial veins into the left atrium and through Thebesian veins into the LV, with concomitant increase of LV end-diastolic pressure. At worst, stagnation of blood within dilated left cardiac chambers may favor the formation of clots and induce pulmonary vein thrombosis [33].

Pulmonary congestion develops consecutively to the passive upstream transmission of elevated LV pressure [34]. Lung extravascular water accumulation is potentiated by the increased vascular permeability in the context of VA-ECMO-induced SIRS. The magnitude of afterload increase, LV distension, and pulmonary congestion is dependent on several parameters, including VA-ECMO flow, systemic vascular resistance, and LV residual function [31, 35].

Pulmonary congestion may jeopardize lung parenchymal cell oxygenation through two mechanisms. Firstly, interstitial edema increases the thickness of the alveolar-capillary barrier, hence the diffusion distance for oxygen between alveoli and parenchymal cells, whose oxygenation primarily depends on oxygen diffusing from alveolar spaces [36]. Secondly, alveolar edema results in a marked reduction of local alveolar PO_2_ (PAO_2_). Alveolar epithelial cells, normally exposed to PAO_2_ above 100 mmHg, are sensitive to hypoxia from PAO_2_ below 50 mmHg, which may occur in alveoli flooded by pulmonary edema [37]. Alveolar hypoxia can destabilize intercellular junctions, impair barrier permeability, impede alveolar fluid clearance and surfactant production by pneumocytes, induce local vasoconstriction and neoangiogenesis, and finally trigger local and systemic inflammation [37–39]. Therefore, pulmonary congestion during VA-ECMO creates a vicious circle in which VA-ECMO-induced SIRS and LV pressure overload promote pulmonary edema, leading to alveolar hypoxia which maintains SIRS [37]. Alveolar hemorrhages are another frequent consequence of the combination of pulmonary congestion and the requirement of anticoagulation during ECLS. Even if massive hemoptysis is rare [40], local alveolar hemorrhages are frequent and sustain local inflammatory changes [41, 42].

### Evaluation of pulmonary congestion and cardiac overload during VA-ECMO

Chest X-ray is the simplest exam to assess pulmonary congestion, although its interpretation is complicated by frequently associated abnormalities, such as pneumonia, atelectasis, or alveolar hemorrhages. Chest ultrasound is an effective and reliable alternative method to assess interstitial edema, pleural effusion, and parenchymal consolidation [43]. Echocardiographic examination is mandatory, as it may show left heart dilation and indirect signs of cardiac congestion, such as spontaneous contrast echoes or the presence of “sludge” in heart chambers, as well as the absence of aortic valve opening [44]. Hemodynamic monitoring using pulmonary artery catheter (PAC) has been associated with improved survival in cardiogenic shock [45], notably in the context of mechanical cardiac support. PAC is particularly helpful to identify patients with cardiac distension, by demonstrating elevated left-sided filling pressure [46]. It has been shown that combining a value of pulmonary artery diastolic pressure > 25 mmHg (as a surrogate of pulmonary capillary wedge pressure) with evidence of pulmonary edema on chest X-ray could identify patients with subclinical LV distension [47]. Although these data need further validation, PAC is now advocated by most experts to help manage patients under VA-ECMO [48].

### Strategies to reduce pulmonary congestion during VA-ECMO

Severe pulmonary congestion during VA-ECMO is associated with a dismal prognosis, and its treatment is mandatory [5, 49]. Inotropic agents increase cardiac contractility, promote aortic valve opening, and reduce LV dilation and filling pressure. Reducing VA-ECMO flow to decrease LV afterload, as long as residual LV ejection is present and peripheral perfusion maintained, should also be considered. The insertion of an intra-aortic balloon pump (IAPB) is a further option to decrease LV afterload. As demonstrated by Bréchot et al., IABP in combination with VA-ECMO versus VA-ECMO alone is independently associated with less frequent hydrostatic pulmonary edema and a shorter duration of mechanical ventilation [50]. A recent meta-analysis found concomitant IABP to reduce in-hospital death and length of stay [51].

If previous steps fail to reduce pulmonary edema, the left heart chambers must be directly unloaded (“vented”), either by percutaneous atrial transseptal approach or by using a venting cannula inserted into the left atrium or the LV apex by surgical or trans-aortic approach [52]. In addition, the catheter-mounted microaxial pump Impella® (Abiomed, Danvers, MA) may represent a further efficient device to permit LV unloading [52]. Eliet et al. have recently observed that Impella® not only decreases LV diastolic diameter but also increases pulmonary flow [53]. These different modalities of cardiac unloading during VA-ECMO have been the matter of several extensive recent reviews [52, 54].

## VA-ECMO-related lung ischemia

### Lung blood supply

The lung is characterized by a dual circulation, comprising the pulmonary circulation, which supplies the alveoli for gas exchange, and the bronchial circulation, which conveys oxygen and nutrients to the airways, but not alveoli, whose oxygen supply is almost exclusively provided by direct diffusion from the alveolar spaces [39]. Bronchopulmonary anastomoses allow collateralization between these two circulations. In case of chronic decrease of pulmonary blood flow (e.g., in chronic thromboembolic disease or pulmonary stenosis), the bronchial flow may increase from 1 to 30% of the cardiac output, permitting to compensate this decrease and participate to gas exchange, providing a kind of “rescue flow” to the ischemic areas [55, 56].

### Disturbances induced by VA-ECMO

As depicted in Fig. 1, venous blood during VA-ECMO is derived from the vena cava and the right atrium through the venous canula, resulting in a reduction of right ventricle (RV) filling, pulmonary blood flow, and pulmonary arterial pulsatility [31]. In a porcine model, Vardi et al. demonstrated that the pulmonary capillary blood flow decreases dramatically as the VA-ECMO flow increases [57]. Moreover, in case of pulmonary congestion (see above), the upstream transmission of increased left atrial pressure reduces the transpulmonary perfusion gradient. Ventilation with high positive end-expiratory pressure (PEEP) might also impede pulmonary blood flow by compression of alveolar vessels [58]. Several additional mechanisms, including alveolar hypoxia, reduction of local NO production, and the actions of inflammatory mediators can promote vasoconstriction and the subsequent increase of pulmonary vascular resistance, with a reduction of pulmonary blood flow [59]. It is also noteworthy that blood flow through the bronchial arteries (BAs) is also reduced during VA-ECMO, due to attenuated pulsatility of the systemic circulation (which supplies the BAs). This can further limit blood supply to ischemic areas within the congested lung [60]. Eventually, these various hemodynamic changes may lead to hypoperfusion of the entire pulmonary vasculature, which, superimposed to alveolar hypoxia, can promote a state of global, persistent lung ischemia.

### Strategies to reduce lung ischemia

The best way to overcome such alterations is, of course, the withdrawal of VA-ECMO. If this is not possible, VA-ECMO flow may be reduced to maintain partial pulmonary perfusion. In early experimental studies in pigs, prolonged (18 h) ECMO at full support (with no residual pulmonary blood flow) promoted massive pulmonary parenchymal damage [61], which was not observed at a residual pulmonary blood flow reaching 25% of the systemic cardiac output [62]. An additional strategy relies in the upgrading of VA-ECMO to a hybrid system of veno-veno-arterial support, with an additional cannula inserted into the jugular vein, which provides oxygenated blood within the pulmonary arteries. This approach is sometimes used to treat the Harlequin syndrome (see below), but has not yet been evaluated to prevent lung injury during VA-ECMO.

## Structural lung parenchymal changes

Although no dedicated study has specifically focused on pulmonary histological consequences of VA-ECMO, data from animal models and small human necropsy series have reported several pathological alterations. Koul et al. maintained 6 pigs under total CPB for 18 h before weaning. All the animals died within the next 4 h, and on histological examination, more than 80% of the pulmonary parenchyma displayed edema, hyaline membranes, alveolar hemorrhages, thrombi, and focal necrotic changes [61]. In another experimental study exploring the effects of long-term VA-ECMO without anticoagulation, Mizuno et al. succeeded to maintain a goat up to 5 months under VA-ECMO with a pulmonary blood flow reduced to 40%. At autopsy, diffuse interstitial fibrosis and swelling of endothelial cells with thickening of their basal membrane were noted [63].

In humans, Ratliff et al. reported postmortem findings in 4 patients undergoing VA-ECMO for 7 to 12 days. In two patients, diffuse lung fibrosis was noted, together with liquefaction necrosis of the lower lobes. The authors hypothesized that the combination of an increase in metabolically active cell mass together with partial pulmonary shunting concurred to establish ischemic areas with subsequent necrosis [64]. In an autopsy series of 23 infants supported by VA-ECMO, Chou et al. reported hyaline membrane formation, interstitial and intra-alveolar hemorrhages, and reactive hyperplasia of epithelial and smooth muscle cells, developing already after 2 to 3 days of VA-ECMO support, whereas interstitial fibrosis was noted beyond 7 days [41].

To sum up, VA-ECMO appears mostly associated with signs of protein-rich edema, alveolar hemorrhages, tissue necrosis, and fibrosis, which are reminiscent of the damage noted in the acute respiratory distress syndrome. These changes are likely the result of the combination of inflammatory injury, pulmonary congestion, and hypoxia, with the progressive development of epithelial-endothelial injury, increased vascular permeability, and interstitial collagen deposition [65]. Furthermore, some degree of angiogenesis and vascular remodeling may also play some role, as alveolar hypoxia and chronic ischemia (typical of long-lasting VA-ECMO) can activate several pro-angiogenic cascades in alveolar cells, relying on the hypoxia-inducible factor family or the resistin-like molecule-α [37]. Such alterations could result in long-term changes in pulmonary vascular physiology, with possible detrimental consequences on the right ventricle.

## Potential clinical consequences

### Pulmonary dysfunction during VA-ECMO

The impaired pulmonary function induced by VA-ECMO may require long-lasting mechanical ventilation (MV) which may further alter the lung through ventilator-induced lung injury (VILI). Although there is presently no consensus regarding optimal ventilator settings for MV during VA-ECMO, the principles of lung-protective ventilation should be applied [66].

Furthermore, prolonged MV increases the risk of ventilator-associated pneumonia (VAP), which occurs in up to 74% of patients under ECMO, as recently reviewed [67], with risk factors including an age > 65 years, a higher SOFA score on admission, and a history of COPD or hypertension [68]. Causative microorganisms comprise primarily Gram-negative bacilli, with *Pseudomonas aeruginosa* isolated in 18–25% of cases [67]. Diagnosis of VAP may be particularly troublesome, as the usual criteria of VAP are difficult to interpret in the setting of ECMO, and a high clinical index of suspicion coupled to early microbiological sampling are major clues to diagnosis [67]. Treatment of VAP on ECMO is challenging, notably because of the alterations of antibiotic pharmacokinetics occurring in this setting, and frequent therapeutic drug monitoring is therefore recommended [69].

Preventive measures to reduce the risk of VAP include primarily the reduction of MV duration. In this regard, a strategy of early extubation and awake ECMO support is emerging as a promising strategy [70]. In properly selected patients, such strategy not only significantly reduces the incidence of VAP [70], but also permits active mobilization, reduces the overall rate of complications, and increases survival [71].

**Fig. 2:**
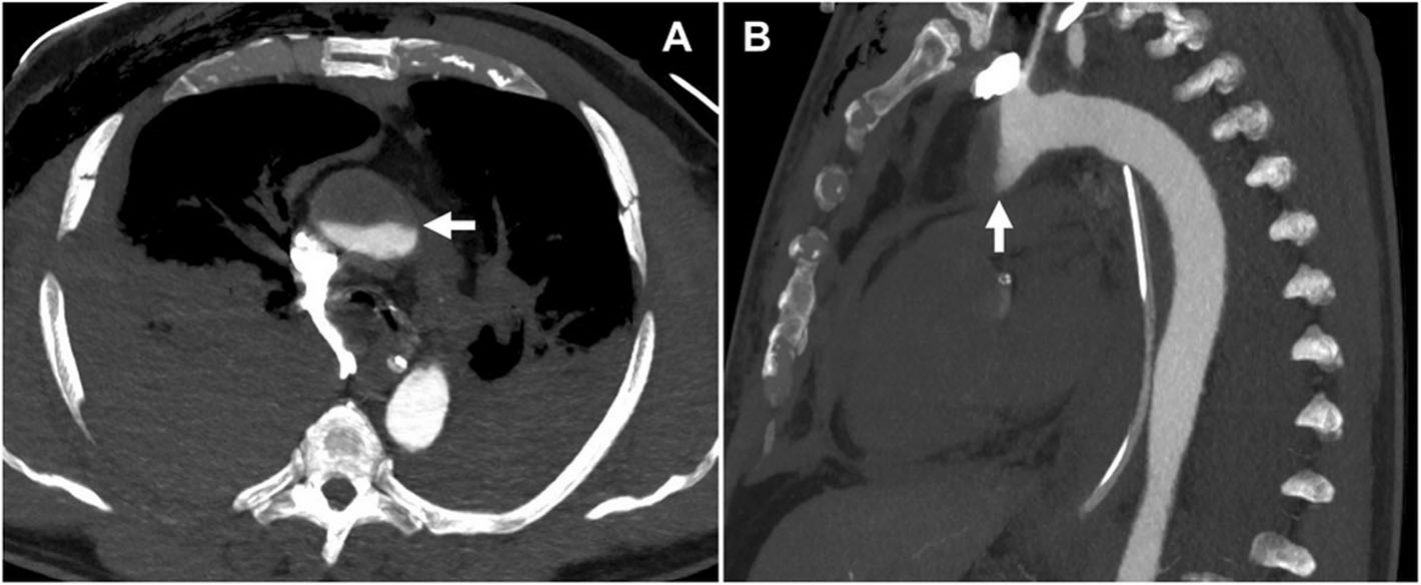
The watershed. **a** Axial. **b** Sagittal. Contrast in the aorta indicates blood flow from the VA-ECMO arterial cannula, whereas absence of contrast within the ascending aorta indicates blood flow from the native heart. The level of blood mixing in the thoracic aorta represents the VA-ECMO watershed (arrows)

**Fig. 3:**
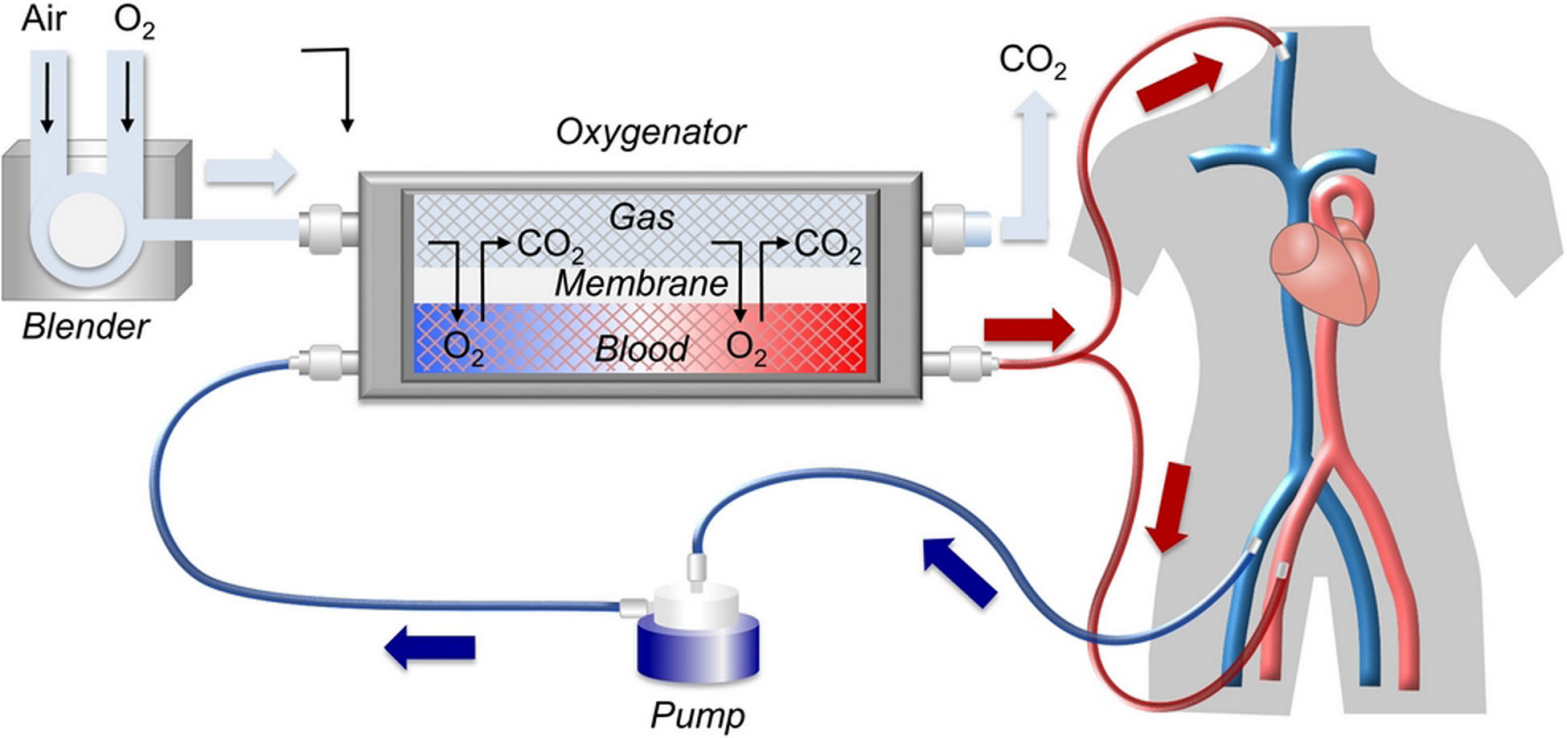
Veno-veno-arterial ECMO. Oxygenated blood is propelled through both the femoral arterial cannula and the additional jugular cannula providing oxygenated blood directly into the right-heart chambers and consequently into the left atrium. This setting permits to wean progressively the arterial cannula in order to switch to veno-venous ECMO

The prototypical consequence of VA-ECMO-dependent impairment of lung function is the development of the “Harlequin syndrome,” reflecting the opposing flows from the heart (antegrade, poorly oxygenated blood flow) and from the peripheral ECMO (retrograde, highly oxygenated blood flow), resulting in differential hypoxia (upper body hypoxemia, lower body normo/hyperoxemia). The level of mixing of the two flows within the aorta is termed the “watershed,” which can be identified in contrast-enhanced CT scan of the chest (Fig. 2). The Harlequin syndrome may be treated by increasing VA-ECMO flow or adding a venous injection cannula either as a hybrid ECMO (veno-veno-arterial ECMO, Fig. 3) or as pure veno-venous ECMO if the function of the heart allows withdrawal of the arterial cannula. Another option consists of switching the arterial cannulation site from femoral to axillary or central (aorta) location, in order to avoid the retrograde flow from the peripheral femoral cannula [72].

Finally, bronchoscopy with bronchial hygiene may be considered routinely in order to maximize chances of successful weaning from VA-ECMO when cardiac function recovers [73].

### Pulmonary dysfunction after weaning from VA-ECMO

VA-ECMO-induced lung alterations may only appear after weaning and the restoration of physiological pulmonary artery blood flow. In a series of 55 patients who underwent long-term mechanical assist device implantation under VA-ECMO (L-VAD, bi-VAD, or total artificial heart), Boulate et al. noticed that 27% of patients develop acute lung injury (ALI) few hours after restoration of pulmonary blood flow, with a significant impact on mortality. The authors hypothesized that chronic lung ischemia during VA-ECMO support could promote alveolar frailty and that the sudden restoration of an antegrade pulsatile pulmonary blood flow creates a massive pulmonary bed overload responsible of ALI [74]. Accordingly, one of the identified risk factors of ALI in this study was the occurrence of a pulmonary edema during the week preceding the implantation of the mechanical device, featuring a preexisting lung frailty.

This form of ALI is reminiscent of reperfusion pulmonary edema, a well-known and described condition that occurs after reperfusion of a chronic low pulmonary blood flow situation, as in correction of tetralogy of Fallot [75] or pulmonary endarteriectomy for chronic embolism [76]. Such reperfusion pulmonary edema relies both on ischemia-induced chronic inflammation and reperfusion injury that involves similar mechanisms than those described above [77].

In order to help the decision-making process in patients under VA-ECMO, Chen et al. developed a risk factor-calling score (RFSS) to select patients eligible for L-VAD or HTX. The RFSS has 5 items and 16 points, 7 of which are allocated to pulmonary dysfunction. A RFSS > 7 predicted a poor outcome, which emphasizes the relative burden of pulmonary dysfunction in the outcome of patients under VA-ECMO in a bridge strategy [78].

### Long-term outcome

It is currently unknown whether lung damage and dysfunction induced by VA-ECMO have an impact on long-term outcome, the more so that many unrelated factors may interfere with such outcome, such as prolonged ICU stay, previous health condition, or reduced LV ejection fraction. A few studies focused on long-term health-related quality of life (HRQL) in VA-ECMO survivors after cardiogenic shock. Combes et al. questioned 28 VA-ECMO survivors about their HRQL via the short-form 36 questionnaire (SF-36). Mean VA-ECMO duration and follow-up were respectively 7 days (5 to 10) and 11 months (3 to 39). In comparison to sex- and age-matched controls, VA-ECMO survivors disclosed significantly lower role-physical score and a trend to a lower physical function, even though most patients recovered a good cardiac function with mean LVEF 51% or underwent HTX [79]. These results were confirmed by other studies showing lower SF-36 values of physical functioning and role-physical scores in VA-ECMO survivors compared to standard population [80, 81]. These data however do not give any information with respect to the potential long-term burden of pulmonary alterations associated with VA-ECMO, and future studies should be designed to address this issue, for example by performing delayed lung functional tests in long-term survivors of VA-ECMO.

## Conclusion

VA-ECMO elicits several pathophysiological disturbances which may significantly impact on lung integrity and function. First, the rapid development of a systemic inflammatory response with pulmonary involvement is an unavoidable consequence of the artificial VA-ECMO circuitry. Second, due to retrograde blood flow within the thoracic aorta, peripheral VA-ECMO has the propensity to increase LV afterload, which may favor the congestion of alveoli already affected by the ongoing inflammation.

Third, persistent lung ischemia due to the partial shunting of the pulmonary circulation and reduced pulsatility of the bronchial circulation may elicit further cytotoxicity within the whole lung parenchyma. Limited evidence from human observational studies and animal models indicates that VA-ECMO support for more than a few days may lead to severe structural changes of the lung parenchyma and interstitial fibrosis, which could result in long-term functional limitation. Clinicians in charge of VA-ECMO patients should be aware of its effects on lung physiology and should take all measures to limit such consequences, including the maintenance of VA-ECMO support as short as possible, the early diagnosis and treatment of cardiac overload and pulmonary congestion, and the application of lung-protective ventilation. Future studies specifically addressing the issue of the pulmonary consequences of VA-ECMO are warranted.

## Data Availability

Not applicable.

## Abbreviations

ALI: Acute lung injury
ARDS: Acute respiratory distress syndrome
CARS: Compensatory anti-inflammatory response syndrome
CPB: Cardio-pulmonary bypass
CS: Contact system
EC: Endothelial cell
HTX: Heart transplantation
IABP: Intra-aortic balloon pump
ICU: Intensive care unit
IL: Interleukine
LA: Left atrium
LV: Left ventricle
MMP: Matrix metalloproteinase
MI: Myocardial infarction
MV: Mechanical ventilation
PA: Pulmonary artery
PAF: Platelet-activating factor
PEEP: Positive end expiratory pressure
PMN: Polymorphonuclear
PV: Pulmonary vein
RA: Right atrium
ROS: Reactive oxygen species
RV: Right ventricle
SIRS: Systemic inflammatory response syndrome
TF: Tissue factor
TNF: Tumor necrosis factor
TXA: Thromboxane
VA-ECMO: Veno-arterial extracorporeal membrane oxygenation
VAD: Ventricular assist device
VAP: Ventilator-associated pneumonia
VILI: Ventilator-induced lung injury
